# Moyamoya disease with Sjogren disease and autoimmune thyroiditis presenting with left intracranial hemorrhage after messenger RNA-1273 vaccination: A case report

**DOI:** 10.1097/MD.0000000000028756

**Published:** 2022-02-11

**Authors:** Yi-Hsin Lin, Hsuan Huang, Wen-Zern Hwang

**Affiliations:** a Division of Endocrinology and Metabolism, Department of Internal Medicine, Taiwan Adventist Hospital, Taipei, Taiwan; b Division of Pediatric Surgery, Department of Surgery, Mackay Memorial Hospital, Taipei, Taiwan; c Division of Neurosurgery, Department of Surgery, Taiwan Adventist Hospital, Taipei, Taiwan.

**Keywords:** autoimmune disease, brain hemorrhage, COVID-19 vaccine, moyamoya

## Abstract

**Rationale:**

The new vaccines are emergently authorized and currently approved for use to protect against the coronavirus disease 2019 (COVID-19) pandemic and serious adverse events are uncommon. Moyamoya disease (MMD) with autoimmune disease is a rare entity and usually presents with intracranial hemorrhage in adults.

**Patient concerns:**

We reported a 40-year-old female patient with Sjogren disease and autoimmune thyroiditis, who had received the second dose of Moderna (mRNA-1273) vaccination. Three days later, she presented with left intraventricular and intracerebral hemorrhage as a complication.

**Diagnosis:**

After a series of diagnostic workups, left intracranial hemorrhage was associated with MMD.

**Interventions:**

Emergent external ventricular drainage and subsequent stereotactic evacuation of hematoma with insertion of intracranial pressure monitoring were performed.

**Outcomes:**

Under the care of the neurocritical care team, her physical condition improved gradually. The neurological sequelae was noted by defects of cognitive function, apraxia, agnosia, and impaired executive function. She was discharged after eight weeks with a follow-up in the vascular neurology clinic planning for performing revascularization.

**Lessons:**

To the best of our knowledge, no similar case has been reported before, and this is the first case of MMD complicated with intracerebral and intraventricular hemorrhage after mRNA-1273 vaccination. It is noticeable to assess the vaccine safety surveillance and raise the alertness about moyamoya in patients with autoimmune diseases during the COVID-19 pandemic. Further studies for risk evaluation of COVID-19 vaccines in patients with autoimmune diseases might be required in the future.

## 1 Introduction

Severe acute respiratory syndrome coronavirus 2 (SARS-CoV-2) spread globally, that caused significant morbidity and mortality of the coronavirus disease 2019 (COVID-19) in recent 2 years.^[1]^ Highly effective vaccines have been developed and produced rapidly with the use of diverse technologies to increase population immunity and prevent severe disease. No major safety warnings, other than rare cases of anaphylaxis, were reported in the initial trials of SARS-CoV-2 vaccines, which involved tens of thousands of adults, and the risk of serious adverse events was remarkably low after the vaccination of more than 400 million people worldwide to date.^[2]^ Since the early May of 2021, a large-scale community outbreak of COVID-19 erupted in Taiwan, Central Epidemic Command Center in Taiwan prompts the public vaccination against COVID-19, including 2 different kinds of vaccines, the ChAdOx1 nCoV-19 (AZD1222), a recombinant chimpanzee adenoviral vector encoding the spike protein of SARS-CoV-2, and the messenger RNA (mRNA)-based vaccine produced by Moderna (mRNA-1273).^[3]^ Like any vaccine, SARS-CoV-2 vaccines can induce local (including pain, redness, or swelling at the site of injection) or systemic (including fever, fatigue, headache, myalgia, arthralgia, chills, diarrhea, nausea, or vomiting) adverse drug reactions (ADRs). Most reactions to vaccines are mild to moderate and go away within a few days on their own. More serious or long-lasting ADRs are possible but extremely rare. It is worthy to note that more people experienced the ADRs after the second dose than after the first dose of the Moderna vaccine.^[4]^ In contrast to the ChAdOx1 nCoV-19 vaccine, adverse reactions after the second dose were reported milder and less frequent.^[5]^ Thus, vaccination providers and recipients need to expect that there may be some side effects after either dose of different vaccines.

Typical moyamoya disease (MMD) is a rare cerebrovascular disease, which is characterized by bilateral progressive steno-occlusion of the distal internal carotid arteries (ICAs) or proximal middle cerebral arteries and anterior cerebral arteries with the abnormal formation of small collateral vessels at the level of the circle of Willis.^[6]^ These small collateral vessels, called moyamoya vessels, produce a characteristic smoky appearance on angiography, like a puff of smoke in the air, giving a name “
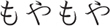
 (moyamoya)” in Japanese. The small collateral vessels are weak and prone to bleeding, microaneurysm, and thrombosis. MMD was first described in Japan in 1957, and it is more commonly reported in East Asian countries than elsewhere.^[7,8]^ The estimated incidence of the disease shows regional differences with 0.35 to 0.94 per 100,000 in Japan, 2.3 per 100,000 in South Korea, and 0.57 per 100,000 in the United States. It occurs most frequently in females, and bimodal peak incidences are among persons aged 1 to 10 in the majority, then followed by aged 30 to 40.^[8,9]^

We reported 1 female patient who had received the second dose of Moderna (mRNA-1273) vaccination. Three days later, she presented with left intraventricular hemorrhage (IVH) and intracerebral hemorrhage (ICH) as a complication, which was associated with MMD after a series of diagnostic workups. In September 2021, we performed an English literature search by using the keywords “ brain hemorrhage, covid-19, vaccine, moyamoya” in PubMed and Google databases. To the best of our knowledge, no similar case has been reported previously, and this is the first MMD case complicated with IVH and ICH after mRNA-1273 vaccination. It calls for safety surveillance of the mRNA vaccine and the alertness about moyamoya in patients with autoimmune diseases during the COVID-19 pandemic.

## 2 Consent for publication

Written informed consent was obtained from the patient for publication of this case report and accompanying images. This case report was conducted under the Declaration of Helsinki. It was approved by the Institutional Review Board of Taiwan Adventist Hospital (Protocol No: 110-E-10).

## 3 Case report

**Figure 1:**
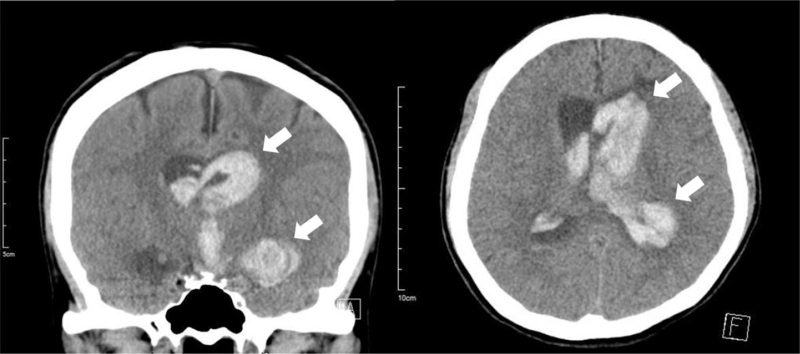
Urgent computed tomography scan of the brain without contrast revealed left caudate nucleus, temporal lobe intraventricular and intracerebral hemorrhage (white arrows) with hydrocephalus.

**Figure 2:**
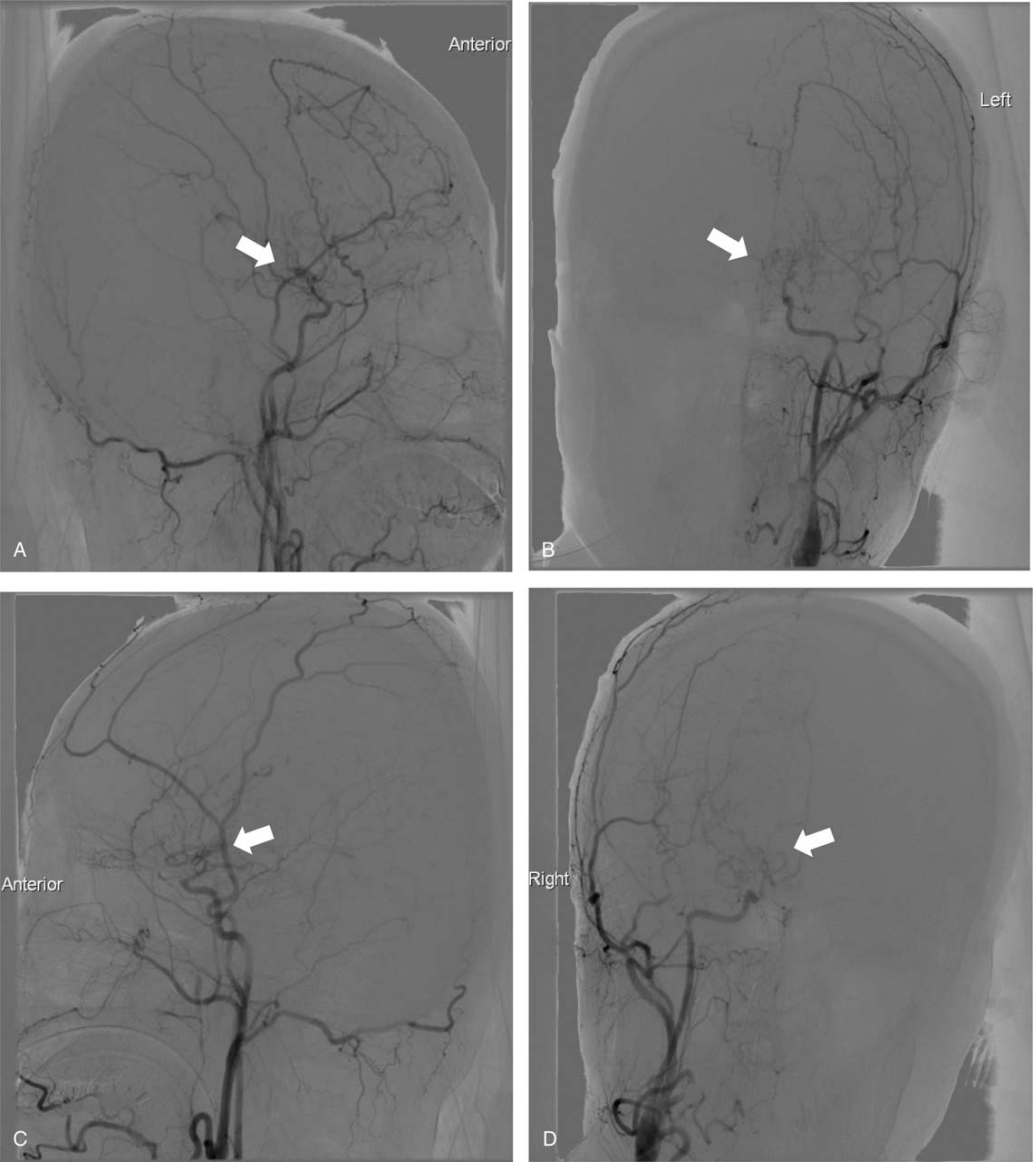
Diagnostic cerebral angiography (A. left lateral view, B. left Towne's view, C. right lateral view, D. right Towne's view) revealed the bilateral distal internal carotid artery steno-occlusion with the constricted flow in middle cerebral arteries (MCAs) and anterior cerebral arteries (ACAs) with cortical collateralization pattern from the external carotid artery system, consistent with typical moyamoya angiopathy. The white arrows were the minimized cluster of smoggy moyamoya vessels in the skull base (Suzuki staging system as stage V).

**Figure 3:**
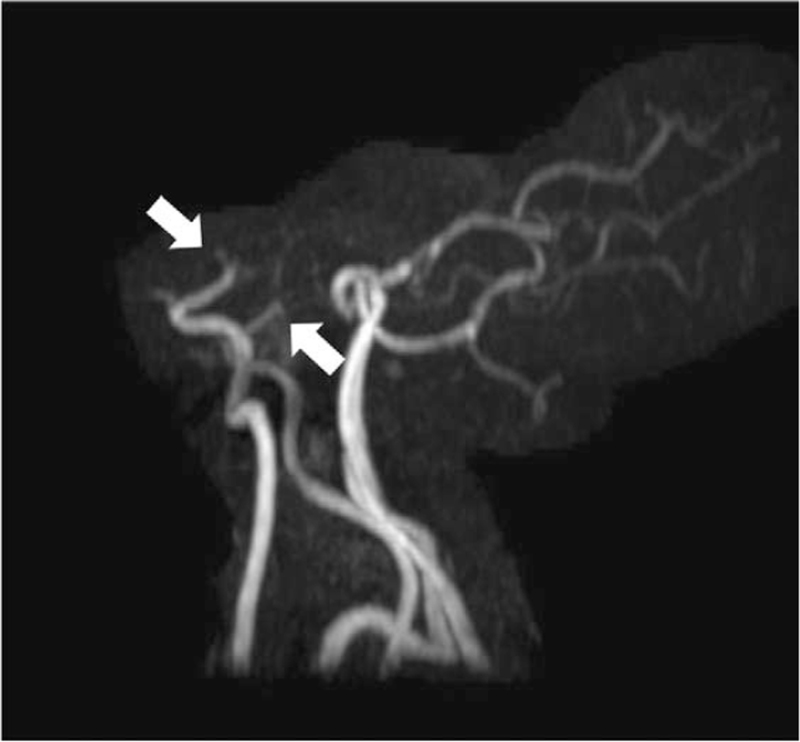
Contrast-enhanced magnetic resonance angiography of the brain (oblique view) revealed bilateral distal internal carotid artery (white arrows) steno-occlusion.

A 40-year-old woman with background history of Sjogren disease and autoimmune thyroiditis, who was under well control of regular medication (azathioprine 12.5 mg once daily, hydroxychloroquine 200 mg twice daily, and propylthiouracil 25 mg once daily). She had received her second dose of Moderna (mRNA-1273) vaccine on July 20, 2021, 28 days after her first dose. For the first 2 days after her second shot, she not only had some local adverse effects, including soreness on the site of injection and the arm, but also had persistent fever around 39 to 40°C with dizziness and headaches which could not be relieved by taking acetaminophen. On day 3 after receiving the vaccine, she suffered from severe headaches with a decreased level of consciousness and a tonic-clonic seizure that was witnessed by her sister. On arrival to the emergency department, she was in a hypertensive urgency condition with the blood pressure up to 185/145 mm Hg, a rapid heart rate of 122 beats per minute, and a respiratory rate of 22 breaths per minute. Her Glasgow coma scale was E2V2M4 and the muscle power of both upper and lower limbs was 3. She was immediately intubated for airway protection. An urgent blood gas analysis revealed pH 7.47, PO_2_ 486.3 mm Hg, PCO_2_ 22.7 mm Hg, HCO_3_ 19.5 mEq/L, with FiO_2_ 100%. The electrocardiography noted sinus tachycardia and the chest X-ray was normal. An urgent computed tomography scan of the brain without contrast revealed the left caudate nucleus, temporal lobe IVH and ICH with hydrocephalus (Fig. 1). The complete blood count, D dimer test, coagulation, and complete metabolic panel initially revealed no abnormality, except for mild leukocytosis (10.6^1000/μL [reference value 3.8–10.0]), elevated fibrinogen level (599.5 mg/dL [reference value 200.0–393.0]), high C-reaction protein (12.4 mg/dL [reference value <1.0]), and positive of Anti-SSA/Ro antibodies (247 Au/mL [reference value >120 for positive]). The anti-platelet factor 4 antibody levels performed twice with the interval of 1 week (122.61, 63.89 ng/mL [reference value <50], optical density value 0.705, 0.387 [reference value <0.4], respectively) were mildly elevated but without clinical significance (Table 1), which could initially exclude the possibilities of central venous sinus thrombosis or vaccine-induced immune thrombotic thrombocytopenia. Emergent external ventricular drainage and subsequent stereotactic evacuation of hematoma with insertion of intracranial pressure monitoring were performed. Three days after the admission, the IVH had much improvement and hydrocephalus had mostly abated. She underwent diagnostic cerebral angiography for further confirmation, which revealed the bilateral distal ICA steno-occlusion with the constricted flow in middle cerebral arteries and anterior cerebral arteries with cortical collateralization pattern from the external carotid artery system that was consistent with typical moyamoya angiopathy (MMA). The minimized cluster of “smoke-like” moyamoya vessels was found at the level of the circle of Willis and the Suzuki staging system was stage V (Fig. 2). Under the care of the neurocritical care team in the intensive care unit for 9 days, her condition improved gradually, and the Glasgow coma scale recovered to E4VEM6. Both the external ventricle drainage tubes and the endotracheal tube were successfully removed. Furthermore, she subsequently underwent a contrast-enhanced magnetic resonance imaging and angiography of the brain, which revealed hemorrhage mostly resolved and bilateral distal ICA steno-occlusion that was compatible with the previous angiography (Fig. 3). Afterward, she received the rehabilitation program and was improving in her physical condition, but the neurological sequelae included defects of cognitive function, apraxia, agnosia, and impaired executive function was noted, which was compatible with the diffuse cortical dysfunction on the electroencephalography. She was discharged after 8 weeks with a follow-up in the vascular neurology clinic planning for performing revascularization.

**Table 1:** Laboratory tests on the arrival and further work-up.

	Result	Reference value
Hemoglobin	12.6 g/dL	12.0–16.0
**White blood cell**	**10.6^1000/μL**	**3.8–10.0**
Platelet	219^1000/μL	140.0–450.0
PT	10.2 s	8.0–12.0
INR	0.98	0.85–1.15
APTT	23.5 s	23.9–35.5
D dimer test	0.44 mg/L FEU	<0.55
**Fibrinogen (nephelometry)**	**599.5 mg/dL**	**200.0–393.0**
Creatinine	0.56 mg/dL	0.6–1.2
S-GPT	11 IU/L	7-52
**C-reaction protein**	**12.4 mg/dL**	**<1.0**
Procalcitonin	0.062 ng/mL	<0.5
Total cholesterol	162 mg/dL	<200
Triglyceride	53 mg/dL	<150
High density lipoprotein-cholesterol	57 mg/dL	≥50
Low density lipoprotein-cholesterol	95.3 mg/dL	<130
HbA1c	5.3%	4.0–6.0
Free T4	1.78 ng/dL	0.66–1.17
TSH	0.486 uIU/mL	0.38–5.33
Anti-TPO	380 IU/mL	<34
Anti-thyroglobulin antibody	566 IUmL	<115
TSH receptor antibody	2.7 IU/L	<1.75
**Anti-SSA/Ro**	**247 Au/mL**	**>120, positive**
Anti-cardiolipin antibody	Negative	Negative
Anti-DNA	2.3 IU/mL	<10 negative
Anti-phospholipid antibody	Negative	Negative
Anti-β2-glycoprotein-I antibody	Negative	Negative
Anti-SSB/La	36 Au/mL	<100
Anti-Smith-Ab	11 Au/mL	<100
Anti-nRNP	15 Au/mL	<100
Anti-SCL70	7 Au/mL	<100
Anti-JO-1	12 Au/mL	<100
Lupus antibody	Negative	Negative
Anti-nuclear antibody	Negative	Negative
Rheumatoid factor	Negative	Negative
Protein S	138.6%	63.5%–149.0%
**Anti-platelet factor 4 antibody**	**First time: 122.61 ng/mL (optical density value 0.705)** **Second time: 63.89 ng/mL (optical density value 0.387)**	**>50 (optical density value 0.4), positive**
COVID-19 RT-PCR	Negative	Negative

Anti-TPO = antithyroid peroxidase antibody, APTT = activated partial thromboplastin Time, COVID-19 = coronavirus disease 2019, HbA1c = hemoglobin A1c, INR = international normalized ratio, PT = prothrombin time, RT-PCR = reverse transcription-polymerase chain reaction, S-GPT = serum-glutamate pyruvate transaminase, T4 = thyroxine, TSH = thyroid stimulating hormone.

## 4 Discussion

MMD corresponds to typical MMA of unknown etiologies and may have genetic susceptibilities but no underlying risk factors.^[9,10]^ Accumulating evidence from the whole genome-wide association study demonstrates that the Ring Finger Protein 213 (*RNF 213*) gene on chromosome 17q25.3 is an important susceptibility factor for familiar MMD patients in several East Asian countries.^[11,12]^ The linkage between familial MMD and chromosomes 3p24.2-p26, 6q25, 8q23, and 12p12 are also reported in other studies.^[13–15]^ Furthermore, Moyamoya syndrome (MMS) refers to atypical MMA associated with underlying acquired (i.e., autoimmune diseases) or inherited (i.e., neurofibromatosis type 1, sickle cell anemia, Down syndrome, Noonan syndrome, Costello syndrome, Alagille syndrome) conditions that may trigger pathophysiologic processes leading to the abnormal angiopathy.^[8,9]^ However, the pathophysiology of MMD and MMS has still not been fully elucidated to date.

MMD and MMS have heterogeneous phenotypes with various clinic presentations depending on different stages and associated conditions. Cerebral angiography is the gold standard for diagnosing MMD and the Suzuki staging system is used to evaluate its progression.^[9]^ The staging system is divided into 6 stages (I–VI), which are based on the progression degree of small vessel collaterals and the compensation of intracranial circulation from the extracranial arterial system. Our patient was diagnosed with typical MMD (Suzuki stage V), which was defined by the disappearance of all the main cerebral arteries arising from the ICA system, and further minimization of the “smoke-like” moyamoya vessels with an increase in the collateral pathways from the external carotid artery system.

Our patient has a history of Sjogren disease and autoimmune thyroiditis. The relationship between moyamoya and autoimmune diseases has been documented.^[9,16–18]^ In some immunohistochemical studies of MMD, deposition of immunoglobulins was found in the intima of the ICA, and T cells infiltrated in subendothelial tissue of the carotid wall.^[6,19]^ Several studies and meta-analyses reported significantly elevated levels of thyroid autoantibodies in patients with MMA than in those without MMA.^[20,21]^ These studies suggest the possible immunological mechanism in the pathogenesis of moyamoya. In addition, the booster vaccination elicited exuberant cytokine activation or cytokine storm, that may interact with the *RNF 213* gene and the endothelial membrane protein (caveolin-1) leading to MMA.^[22,23]^

Moyamoya has varying clinical symptoms including transient ischemic attack, ischemic stroke, hemorrhagic stroke, seizure, headache, and cognitive dysfunction. The incidence of these symptoms depends on the age, regional and ethnic differences of patients. While most pediatric patients with moyamoya are characterized by cerebral ischemia symptoms, adult patients usually present with intracranial hemorrhage. The complicated pathophysiologic processes finally lead to chronic progressive steno-occlusion of the large intracranial arteries around the circle of Willis and the formation of small vessel collaterals, which are weak, and prone to developing thrombosis and microaneurysms. These fragile collaterals and microaneurysms are easily bleeding and ruptured by severe hemodynamic stress on the cerebral circulation system, which is triggered by vigorous exercise, crying, singing, coughing, straining, fever, hyperventilation, dehydration, and systemic hemodynamic change.^[24,25]^ Our patient had received her second dose of the Moderna (mRNA-1273) vaccine, and then she suffered from severe systemic inflammatory response syndrome (SIRS) with a high fever around 39 to 40°C and probably hypertensive crisis. Although the exact pathogenesis was unclear, the potential mechanism might be that severe SIRS precipitated the hypertensive crisis unawareness and the intracranial pressure, which progressed the rupture of fragile MMA and led to IVH and ICH.

The new mRNA-based vaccines, including Moderna, are emergently authorized and currently approved for use to protect against the COVID-19 pandemic. Based on recent phase 3 trial preliminary data, the overall rate of severe systemic ADRs is low, and most acute ADRs are self-limiting.^[4]^ For example, in the trial that used the Moderna (mRNA-1273) vaccine (15,185 vaccine users vs 15,166 non-users), the severity of the systemic ADRs increased after the second dose in the mRNA-1273 group, but the overall incidence of severe ADRs was 0.5%. However, a greater percentage of the participants who received the Moderna vaccine reported fever (15.5%), fatigue (70%), headache (64.7%), myalgia (61.5%), arthralgia (46.4%), chills (45.4%), nausea or vomiting (23%), and pain (92%), redness (10%), or swelling (14%) at the site of injection. Both local and systemic ADRs were more frequent among the young people (18–<65 years of age) than among the elders (≥65 years of age). Based on the literature review, antipyretics might somewhat reduce vaccine immune responses.^[26,27]^ It is recommended that antipyretics can be used as over-the-counter medications to ameliorate local or systemic ADRs postvaccination, however, the prophylactic use of antipyretics is not recommended before or at the time of vaccination. Prophylactic use of antipyretics to control ADRs may be a dilemma in patients with MMA, especially in our patient with the unknown underlying MMA.

## 5 Conclusion

MMS related to autoimmune disease is a rare entity. The immunological mechanism plays the role of pathogenesis in this disease. Postvaccination exuberant cytokine activation and severe SIRS both act as the precipitating factors for the progression of MMA. It is noticeable to assess the vaccine safety surveillance and raise the alertness about moyamoya in patients with autoimmune diseases during the COVID-19 pandemic. Further studies for risk evaluation of COVID-19 vaccines in patients with autoimmune diseases might be required in the future.

## Acknowledgments

The authors would like to express their deepest gratitude to all the people who participated in this study. Our heartfelt thanks go to the hospital officials for their guidance and support for this study.

## Author contributions

**Data curation:** Yi-Hsin Lin.

**Investigation:** Yi-Hsin Lin, Hsuan Huang.

**Resources:** Wen-Zern Hwang.

**Writing – original draft:** Yi-Hsin Lin, Hsuan Huang.

**Writing – review & editing:** Yi-Hsin Lin.
